# Mitochondrial phylogenomics of Hemiptera reveals adaptive innovations driving the diversification of true bugs

**DOI:** 10.1098/rspb.2017.1223

**Published:** 2017-09-06

**Authors:** Hu Li, John M. Leavengood, Eric G. Chapman, Daniel Burkhardt, Fan Song, Pei Jiang, Jinpeng Liu, Xuguo Zhou, Wanzhi Cai

**Affiliations:** 1 Key Laboratory of Pest Monitoring and Green Management, Ministry of Agriculture, Department of Entomology, China Agricultural University, Beijing 100193, People's Republic of China; 2 Department of Entomology, University of Kentucky, Lexington, KY 40546, USA; 3 Naturhistorisches Museum, Augustinergasse 2, 4001 Basel, Switzerland; 4 Markey Cancer Center, University of Kentucky, Lexington, KY 40536, USA

**Keywords:** Hemiptera, phylogeny, evolutionary history, ancestral character state reconstruction, mitochondrial genome

## Abstract

Hemiptera, the largest non-holometabolous order of insects, represents approximately 7% of metazoan diversity. With extraordinary life histories and highly specialized morphological adaptations, hemipterans have exploited diverse habitats and food sources through approximately 300 Myr of evolution. To elucidate the phylogeny and evolutionary history of Hemiptera, we carried out the most comprehensive mitogenomics analysis on the richest taxon sampling to date covering all the suborders and infraorders, including 34 newly sequenced and 94 published mitogenomes. With optimized branch length and sequence heterogeneity, Bayesian analyses using a site-heterogeneous mixture model resolved the higher-level hemipteran phylogeny as (Sternorrhyncha, (Auchenorrhyncha, (Coleorrhyncha, Heteroptera))). Ancestral character state reconstruction and divergence time estimation suggest that the success of true bugs (Heteroptera) is probably due to angiosperm coevolution, but key adaptive innovations (e.g. prognathous mouthpart, predatory behaviour, and haemelytron) facilitated multiple independent shifts among diverse feeding habits and multiple independent colonizations of aquatic habitats.

## 1. Introduction

Ernst Mayr defined evolutionary novelty as ‘any newly acquired structure or property that permits the performance of a new function, which, in turn, will open a new adaptive zone’ [1]. Driven by adaptive modifications and the colonization of new ecospaces, evolutionary radiations of animals and plants have long been recognized as driving today's biodiversity. Tracking the evolutionary origins of morphological novelty has fascinated biologists for over a century [2]. Even though stochastic factors lead to the development of new lineages, only a fraction of these have successfully diversified over time. Some of the major Metazoan radiations, such as true flies [3] and beetles [4], have been well documented; however, other mega-diverse invertebrate clades have not received the attention they deserve.

With an estimated 97 000–103 590 known species [5,6], Hemiptera represents approximately 7% of metazoan diversity. The biodiversity of Hemiptera includes, but is not limited to, plant lice, cicadas, planthoppers, moss bugs, and true bugs. Heteroptera (true bugs) has evolved diverse life histories and specialized morphological adaptations enabling them to colonize both terrestrial and aquatic habitats, and to exploit various food sources ranging from plants, fungi, small arthropods, and vertebrate blood [7]. Although its monophyly is well supported, in particular, by the synapomorphic segmented, piercing-sucking mouthparts with elaborate food and salivary pumps that permit fluid-feeding specializations [6] (see the electronic supplementary material, figure S1*a–d*), the higher-level relationships within Hemiptera have been debated for over two and a half centuries [8–10]. Traditionally, Hemiptera has been categorized into ‘Homoptera’ and Heteroptera, sometimes with ordinal status, based on the presence or absence of a gula [8]. More recently, Hemiptera has been subdivided into four major suborders, Sternorrhyncha (Psylloidea, Aleyrodoidea, Aphidoidea, and Coccoidea) (e.g. electronic supplementary material, figure S1*a*,*b*), Auchenorrhyncha (Cicadomorpha and Fulgoromorpha) (e.g. electronic supplementary material, figure S1*c*), Coleorrhyncha (with the only extant family Peloridiidae), and Heteroptera (seven infraorders) (e.g. electronic supplementary material, figure S1*d*,*e*). The sister group relationship between Sternorrhyncha and the remainder of Hemiptera has received strong support from both morphological and molecular evidence [9–12]. However, the monophyly of Auchenorrhyncha has been questioned [10,13,14], and the phylogenetic position of Coleorrhyncha is ambiguous [10,12,14,15]. In addition, relationships among the basal infraorders of Heteroptera are poorly understood [6].

Phylogenetic analysis of Hemiptera based solely on morphology has been challenging. The sedentary lifestyles coupled with phloem-feeding behaviours in some Auchenorrhyncha and especially Sternorrhyncha (behaving as plant parasites) have spurred morphological reductions and losses, neotenous females, extreme sexual dimorphism, and convergently derived morphological characters that would otherwise be useful in phylogenetic analyses [16,17]. The confusion of convergent character states with synapomorphies has contributed to the taxonomic reshufflings of superfamily composition within ‘Homoptera’ [16]. Owing to a large number of morphological features unique to Hemiptera (e.g. the labium forming a sheath for the remaining mouthparts), some of the important characters cannot be readily homologized with structures in the more inclusive groups, resulting in ambiguous or even erroneous ancestral state reconstructions.

Historically, some hemipterists assumed that the ancestor of Hemiptera was phytophagous [18], whereas the ancestor of Heteroptera was considered to be predaceous [19]. The presumed diet of ‘Homoptera’ was intuitive, because the vast majority are plant feeders. The predaceous ancestor of Heteroptera was inferred by the predatory behaviour exhibited by the putative ‘basal’ infraorders, Enicocephalomorpha, Dipsocoromorpha, and Gerromorpha [19]. It is understood that after the Permian-Triassic (P-T) extinction events, many previously exploited niches once again became available for resource partitioning [20]. Heteroptera constitutes approximately 40% of Hemiptera and represents the vast majority of behavioural diversity in terms of diet and habitat. The other three suborders are entirely terrestrial and predominantly phytophagous [7]. Hypotheses of selective forces underlying the diversification of higher-level hemipteran lineages have not yet been substantiated outside of morphology and fossil-based extrapolation [21,22].

With the advent of the Genomics Era, recent analyses have increasingly embraced the molecular resources to advance our understanding of the phylogeny of Hemiptera [10–13,15]. Nevertheless, major issues such as the phylogenetic status (monophyly versus paraphyly) of Auchenorrhyncha and the position of Coleorrhyncha are still unsettled [10,12]. With a recent influx of genomic information, including mitochondrial genomes (mitogenomes), new phylogenetic hypotheses are emerging. Although representing only a subset of the genomic information (approx. 16 000 nucleotides), mitogenomic data have made substantial contributions to resolve intraordinal relationships in insects [3,23,24].

Despite extensive efforts, previous mitogenomic analyses in Hemiptera did not cover all the suborders and infraorders, and had limited resolution due to the substitution saturation and the compositional heterogeneity of mitogenomes [11,13,15]. Here, we sequenced 34 mitogenomes to complement the existing mitogenomic data derived from 94 hemipteran species. Using a holistic sampling approach, we included the mitogenomes from all four suborders and all seven heteropteran infraorders, covering all four superfamilies of Auchenorrhyncha, three of the four superfamilies of Sternorrhyncha (excluding Coccoidea), the only superfamily of Coleorrhyncha, and 19 of the 23 superfamilies of Heteroptera. Using a fossil-calibrated divergence dating analysis, we also carried out the first order-wide diversification study in Hemiptera to track the timing of major cladogenetic events. Equipped with the most comprehensive mitochondrial phylogenomic analysis in Hemiptera and informed by the ancestral state reconstruction of morphological characters, habitat preference, and feeding behaviours, we address the following questions: (i) what is the timing of key morphological adaptations that led to the diversification of habitat utilization and feeding behaviour in Heteroptera? (ii) Was the ancestor of Heteroptera predatory or phytophagous? (iii) What extinction and/or rapid radiation events coincide with the diversification of the major lineages in Hemiptera?

## 2. Material and methods

### (a) Taxon sampling

Previous studies assessed mitochondrial phylogenetic signal limits in Paraneoptera and detected long-branch attraction artefacts among Phthiraptera, Thysanoptera, and Sternorrhyncha [11,25]. Thus, Phthiraptera and Thysanoptera were not included in the taxon sampling of outgroups. We included six outgroup species to represent other paraneopteran lineages as well as the putatively more ancient lineages Blattodea and Mantodea (electronic supplementary material, table S1). As ingroups for phylogenetic analysis, 34 hemipteran species were sequenced in this study, and the sequences of 94 hemipterans were obtained from the National Center for Biotechnology Information (NCBI) database. All 128 mitogenomes represent each of the major hemipteran suborders (with coverage of extant taxa) (electronic supplementary material, table S1).

### (b) Complete mitogenome sequence generation

Specimens of 34 hemipterans were collected in 95–100% ethanol and stored at −20°C in the Entomological Museum of China Agricultural University (Beijing, China). Genomic DNA was extracted from the thoracic muscle tissue using the DNeasy blood and tissue kit (Qiagen) following the animal tissue protocol. Whole mitogenomes were generated by amplification, sequencing, and assembly of overlapping PCR fragments, employing general insect mitochondrial primers (electronic supplementary material, table S2). Species-specific primers were designed based on the sequenced fragments to bridge gaps when general primers failed to produce a usable product. Details of the amplification conditions and sequencing strategies were described in our previous study [26].

### (c) Assembly, annotation, and alignment

Sequences from each genome were assembled into contigs using SEQUENCER v5.1 (Gene Codes, Ann Arbor, MI, USA). Protein-coding genes (PCGs) and rRNA genes were identified using BLAST searches of GenBank and alignment with homologous sequences. The tRNAs were identified with tRNAscan-SE v1.21 [27]. Sequences of each PCG (excluding stop codons) were aligned individually based on codon-based multiple alignments using the MAFFT algorithm implemented in the TranslatorX online platform [28]. Ambiguously aligned sites were removed from the protein alignment before back-translating to nucleotides using GBlocks in TranslatorX with default settings. Sequences of each RNA gene were individually aligned using the MAFFT v7.0 online server with G-INS-i strategy [29] and ambiguously aligned sites were omitted using GBlocks v0.91b [30] with default settings. All alignments were then checked and corrected manually in MEGA v6.0 [31] for quality.

### (d) Phylogenetic analyses

Recent phylogenomic studies have shown the ability of site-heterogeneous models (e.g. CAT-based models) to reduce artefacts resulting from mutational saturation and unequal patterns of substitution, which are major problems when analysing genomic data and ancient events [24,25,32–36]. The heterogeneity of sequence divergence within the dataset (e.g. each codon position of PCG and sequences of RNA genes) was analysed using AliGROOVE [37] with the default sliding window size. Indels in the nucleotide dataset were treated as ambiguity and a BLOSUM62 matrix was used as the default amino acid substitution matrix. To account for the strong sequence heterogeneity of the third codon position of the PCGs found in the results of AliGROOVE analysis (electronic supplementary material, figure S2), three datasets were concatenated for phylogenetic analysis: (i) the AA matrix, including amino acid sequences of the 13 PCGs (total of 3 123 amino acids), (ii) the protein-coding plus RNA gene (PCGRNA) matrix, including all three codon positions of the 13 PCGs, two rRNA genes, and 17 tRNA genes (total of 11 652 bp), (iii) the PCG12RNA matrix, including the first and second codon positions of the 13 PCGs, two rRNA genes, and 17 tRNA genes (total of 8 528 bp). Five tRNAs (Ala, Ile, Met, Gln, and Ser) were not found in many nearly complete mitogenomes and therefore were excluded from our analyses.

Bayesian cross-validation was performed to test the fit of two site-heterogeneous mixture models (CAT and CAT + GTR) and site-homogeneous model (GTR) to our mitogenomic data using PhyloBayes 3.3f [38]. The cross-validation was performed according to the PhyloBayes manual in 10 replicates each with 1 100 cycles and the first 100 cycles being discarded as burn-in. The CAT + GTR model was found to be the best fitting model for all datasets (electronic supplementary material, table S3). We then inferred phylogenies from three datasets using PhyloBayes MPI 1.4f [39], with the CAT + GTR model and a discrete gamma distribution with 4 rate categories. In each individual analysis, two independent chains starting from a random tree were run and a consensus tree was calculated by pooling sampled trees from two independent runs, with all analyses satisfactorily converged (maxdiff less than 0.3). The number of discarding trees (burn-in) was calculated case by case to minimize the maxdiff statistics. All analyses were carried out on the CIPRES Science Gateway (https://www.phylo.org) and at the High Performance Computing Cluster at the University of Kentucky Analytics and Technologies (UKAT).

### (e) Ancestral character state reconstruction

Ancestral states for feeding and living habits and morphological characters were reconstructed in Mesquite v2.75 (http://mesquiteproject.org) with Maximum Likelihood (ML) methods. We based ancestral state reconstruction on the tree from PhyloBayes analysis of the PCGRNA dataset with Heteropterodea (Heteroptera + Coleorrhyncha) constrained to be monophyletic. For the ML optimizations, the ‘Markov k-state 1 parameter model’ (MK1 model in which ‘forward’ and ‘backward’ transition rates are equal) was used. Sources of data for feeding habit, living habitat, mouthpart placement, and the presence of hemelytra are listed in the electronic supplementary material, table S4. To make decisions regarding the significance of ancestral character state reconstructions, we followed the recommendation that ancestral character state estimates with a log-likelihood of two or more units lower than the best state estimate be rejected [40]. For ease of interpretation, likelihoods of ancestral states are reported as proportional likelihoods (PL; scaled to add up to 1, thus expressed as a per cent of total likelihood).

### (f) Divergence time estimation

Recent molecular dating analyses have questioned the adequacy of the uncorrelated models of molecular clock relaxation parameters for estimating divergence times with large phylogenomic datasets [41–43]. Based on Bayes factor comparisons, Lepage *et al*. [41] showed that the autocorrelated models provide a significantly better fit than the uncorrelated gamma model for phylogenomic data. Our divergence time estimates were calculated for the two nucleotide and amino acid datasets using PhyloBayes 3.3f [38], the best fitting relaxed clock models, and the optimal tree used in the analysis of ancestral character state reconstruction. We used Bayes factor (calculated using thermodynamic integration) in PhyloBayes to compare three widely used relaxed models, the autocorrelated Lognormal and Cox–Ingersoll–Ross (CIR) process and uncorrelated gamma multipliers (UGAM) [41]. In PhyloBayes, Bayes factor analysis was conducted by running 10 000 points, sampling every 10 points after a burn-in of 1 000. The uncorrelated UGAM model fell into the same category as the models implemented in BEAST, and this model is shown to fit the data more poorly than two autocorrelated models (CIR and Lognormal). As the Bayes factors for the CIR and Lognormal models were similar (electronic supplementary material, table S5), ‘-auto’ analyses (see PhyloBayes manual) were used to compare these two models. For all molecular clock analyses, a birth–death prior on divergence time and the root age of Hemiptera was constrained to prior 306 to 311 Ma, corresponding to the early Hemiptera fossils from the Moscovian age (e.g. *Aviorrhyncha magnifica* and *Protoprosbole straeleni*) [44]. Additionally, 12 fossil calibrations were used with soft bounds, and the details of these fossil calibrations are provided in electronic supplementary material, table S6. We allocated 10% of the probability mass to lie outside each calibration interval. All calculations were performed by running 20 000 generations and sampled every 10 generations (after a burn-in of 2 000 generations).

## 3. Results and discussion

### (a) Phylogeny of Hemiptera

**Figure 1.**
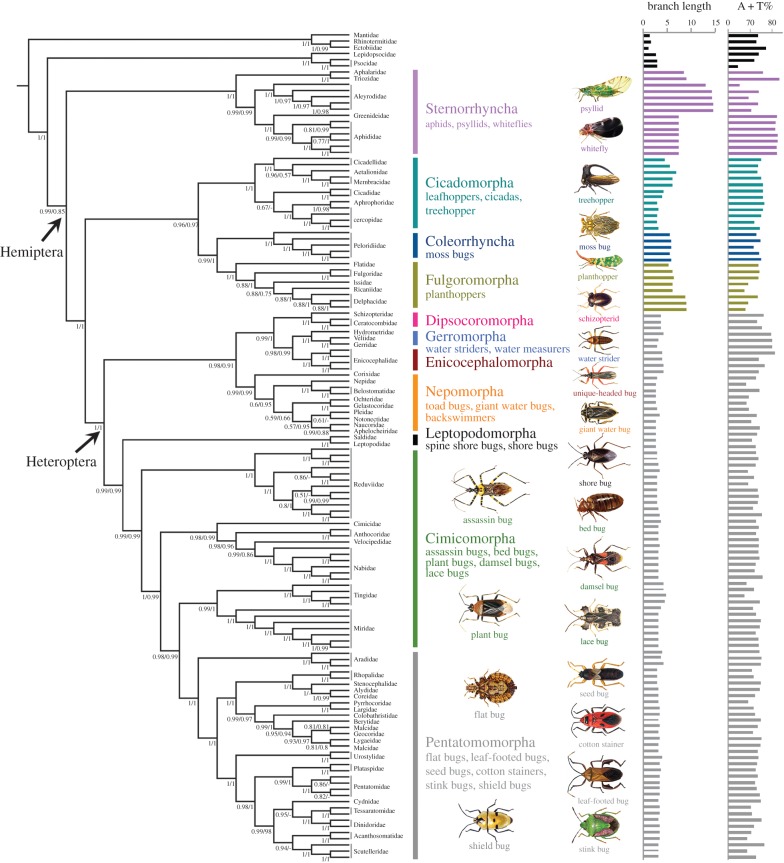
Phylogeny of Hemiptera as inferred from PhyloBayes analyses of the PCGRNA and PCG12RNA datasets under the CAT + GTR mixture model. We show a schematic cladogram depicting the family-level relationships of Hemiptera. Values at nodes are Bayesian posterior probability (PP) using the PCGRNA (left) and PCG12RNA (right) datasets. Dashes indicate PPs less than 0.5. The histogram on the right indicates the branch length of terminal taxa from the Bayesian tree of the PCGRNA dataset and A + T content of terminal taxa from the PCGRNA dataset.

The results of our phylogenetic study based on two nucleotide datasets (PCGRNA and PCG12RNA) produced nearly identical topology with high nodal support values (figure 1; see the electronic supplementary material, figures S3 and S4). The monophyly of Hemiptera was strongly supported, with Sternorrhyncha forming the sister group to all the remaining hemipterans (PP = 1.0 and 0.85). Five long-recognized groups were recovered within Hemiptera: Sternorrhyncha, Cicadomorpha, Fulgoromorpha, Coleorrhyncha, and Heteroptera. However, Auchenorrhyncha was recovered as paraphyletic, with Cicadomorpha forming the sister group to (Fulgoromorpha + Coleorrhyncha). Within Heteroptera, all infraorders were recovered as monophyletic with high support values, except for Cimicomorpha, which was paraphyletic in all analyses. The Cimicomorpha was the closest extant relative of Pentatomomorpha. Leptopodomorpha was recovered as the sister to Cimicomorpha and Pentatomomorpha. The remaining infraorders formed a clade: (Nepomorpha, (Dipsocoromorpha, (Gerromorpha, Enicocephalomorpha))).

**Figure 2.**
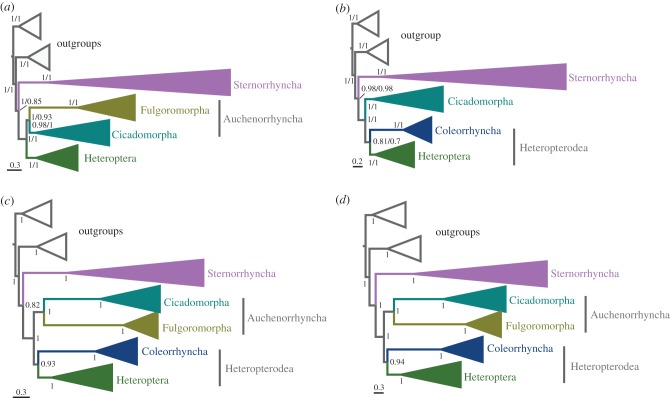
Phylogenetic trees obtained from PhyloBayes analyses of datasets with improved taxon sampling under the CAT + GTR mixture model. (*a*) Datasets with the removal of Coleorrhyncha. (*b*) Datasets with the removal of Fulgoromorpha. In (*a,b*), values at nodes are Bayesian PPs from the modified PCGRNA (left) and PCG12RNA (right) datasets. (*c*) PCG12RNA and (*d*) PCGRNA datasets with the removal of 17 species according to the branch length and the heterogeneity in sequence divergence (see the electronic supplementary material, figure S11). We show a schematic version of the Bayesian trees with some suborders and infraorders collapsed for clarity.

The sister relationship between Sternorrhyncha and all the remaining hemipterans and the monophyletic Auchenorrhyncha have been well resolved in recent studies based on two mitochondrial DNAs and five nuclear loci [10], 1 478 nuclear genes [12], and morphological characters [14]. Mitochondrial phylogenomic analyses, however, suggested that the species with accelerated substitution rates always fall together in one group, e.g. the grouping of Sternorrhyncha with Fulgoromorpha in Song *et al*. [13], Fulgoromorpha with Coleorrhyncha in Cui *et al*. [15], and Sternorrhyncha with Fulgoromorpha and Coleorrhyncha in this study (electronic supplementary material, figure S5). These unexpected groupings were probably caused by the high degree of compositional heterogeneity and, in particular, a significantly accelerated rate in Sternorrhyncha, Fulgoromorpha, and Coleorrhyncha (figure 1; electronic supplementary material, figure S6). The inclusion of rRNA genes in the nucleotide dataset improved the phylogenetic inferences under the site-heterogeneous model, and correctly recovered the majority of deep branches within Hemiptera phylogeny. The sister relationship of Fulgoromorpha and Coleorrhyncha, which exhibited long branches compared with species from Cicadomorpha and Heteroptera, was highly supported. We used the ‘long-branch extraction’ method [45] to sequentially remove Coleorrhyncha and then Fulgoromorpha from the Bayesian analyses of PCGRNA and PCG12RNA datasets using a CAT + GTR model. When coleorrhynchans were excluded, the monophyly of Auchenorrhyncha was recovered in all analyses, with Fulgoromorpha forming the sister group to Cicadomorpha (figure 2*a*; see the electronic supplementary material, figures S7 and S8). When fulgoromorphs were excluded, Coleorrhyncha grouped with Heteroptera (figure 2*b*; see the electronic supplementary material, figures S9 and S10). The four resulting trees showed the identical relationships of heteropteran infraorders as those obtained from the original analyses. These results suggest that the grouping of Fulgoromorpha and Coleorrhyncha is probably an artefact. With the removal of five species with the longest branches in Sternorrhyncha, seven species with the shortest branches in Cicadomorpha, three species with the longest branches in Fulgoromorpha (electronic supplementary material, figure S11*a*), and the two moss bug species in Coleorrhyncha with substantial heterogeneity in their sequence divergence (electronic supplementary material, figure S11*b*), we generated a 117-taxa dataset for the subsequent phylogenetic analysis (see the electronic supplementary material, figure S11). Using PhyloBayes with a CAT + GTR model, the monophyly of Auchenorrhyncha (PP = 1.0) and the sister relationship of Heteroptera and Coleorrhyncha (PP = 0.93 and 0.94) were both recovered by the datasets PCG12RNA (figure 2*c*; electronic supplementary material, figure S12) and PCGRNA (figure 2*d*; electronic supplementary material, figure S13).

With the most comprehensive hemipteran mitogenome sampling to date, the site-heterogeneous mixture model produces an almost fully resolved tree except for the paraphyletic Cimicomorpha. Our results demonstrate that mitogenomes have considerable resolving power in a phylogenetic study because of the ease of sequencing, the feasibility of large taxon sampling, and the use of comprehensive evolutionary models [24,25,32,36].

### (b) Ancestral state reconstructions

**Figure 3.**
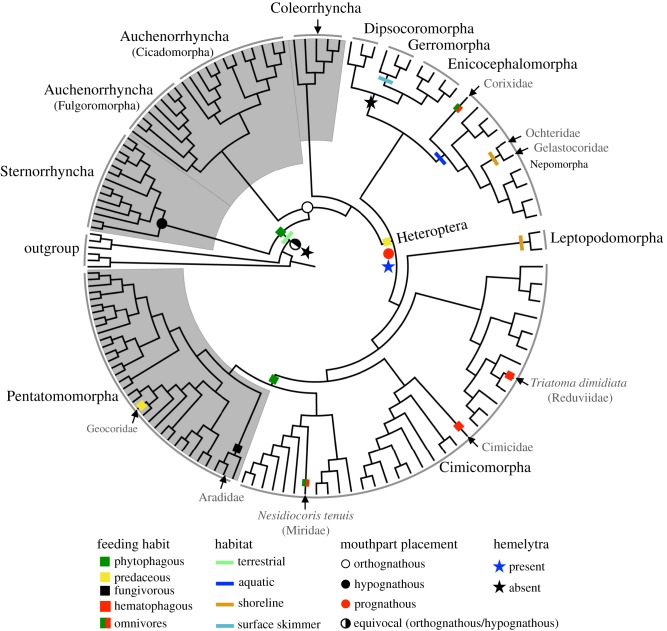
Summary of character state transitions for four characters of hemipteran insects. We based ancestral state reconstruction on the tree from PhyloBayes analysis of the PCGRNA dataset with Heteropterodea (Heteroptera + Coleorrhyncha) constrained to be monophyletic. All character state transitions are judged to be significant by ML methods except where otherwise noted (equivocal or unknown).

Results of ancestral state reconstructions suggest that the common ancestors of Hemiptera, Sternorrhyncha, Auchenorrhyncha, and Coleorrhyncha are all phytophagous and terrestrial with significant PL in all cases, whereas the common ancestor of Heteroptera is predaceous and terrestrial (figure 3; electronic supplementary material, figures S14 and S15). Within Heteroptera, there was a transition from predation to phytophagy in the common ancestor of Miridae + Tingidae (Cimicomorpha, in part) and Pentatomomorpha, and a reversal from phytophagy to predation (Geocoridae). If we include predatory Pentatomidae and Miridae, at least two additional independent reversals would be expected. Omnivory arose twice independently, once from a predaceous ancestor within Nepomorpha (Corixidae) and once from a phytophagous ancestor within Miridae. There were two independent transitions (Reduviidae and Cimicidae) from predation to haematophagy (blood feeding) in Heteroptera. Fungivory in adults arose once from a phytophagous ancestor in Aradidae (and in nymphs of some Auchenorrhyncha). All aquatic, water surface-dwelling, and litter-dwelling infraorders (incidentally all predators except the omnivorous Corixidae) were recovered as a monophyletic group.

There were two independent transitions to shoreline habitat (one from a terrestrial ancestor and one from an aquatic ancestor within Nepomorpha), one transition to surface skimmers from a terrestrial ancestor (Gerromorpha), and one transition from terrestrial to aquatic habitat (Nepomorpha). Optimization of mouthpart origin (see the electronic supplementary material, figure S1*a*–*d*) indicates that the ancestor of Sternorrhyncha had hypognathous mouthparts, the ancestors of Auchenorrhyncha and Coleorrhyncha had orthognathous mouthparts, and the ancestor of Heteroptera had prognathous mouthparts that arose from an ancestor with orthognathous mouthparts (figure 3; electronic supplementary material, figure S16). The presence of hemelytra (electronic supplementary material, figure S1*e*) arose once in the common ancestor of all Heteroptera (figure 3; electronic supplementary material, figure S17). This character state was lost in the common ancestor to Dipsocoromorpha, Enicocephalomorpha, and Gerromorpha.

### (c) Adaptive innovations driving the diversification of true bugs

**Figure 4.**
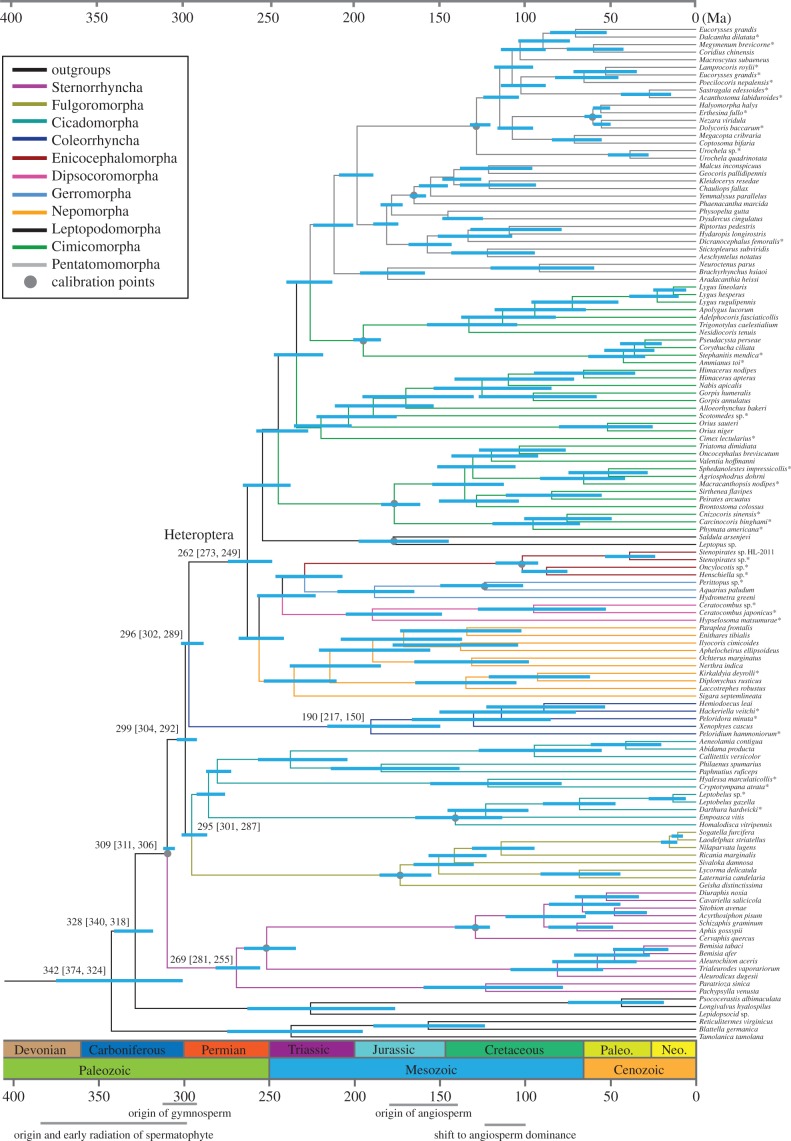
Chronogram showing hemipteran phylogeny and divergence time estimates. Consensus tree presenting divergence dates produced by the PhyloBayes analysis of the PCGRNA dataset (with Heteropterodea, Heteroptera + Coleorrhyncha, constrained to be monophyletic) using 13 fossil calibration points, the CIR autocorrelated process, the site-heterogeneous mixture CAT + GTR substitution model, and soft bound 10%. Blue bars indicate 95% mean confidence intervals of each node. A geological timescale is shown at the bottom. New mitogenomes are highlighted using an asterisk close to the species name. Divergence date estimates based on the PCG12RNA and AA under the CIR model are summarized in the electronic supplementary material, table S7.

Divergence data estimates were not significantly different between datasets using an autocorrelated CIR model (electronic supplementary material, table S7). Hemiptera shares a common ancestor with the remaining Paraneoptera about 328 Ma (confidence interval (CI), 340–318 Ma; figure 4). Subsequently, Hemiptera diversified into Sternorrhyncha and the remaining Hemiptera approximately 309 Ma (CI 311–306 Ma), at the end of the radiation of spermatophytes (seed plants) 385–299 Ma [46]. Our analyses suggest that a Permian diversification of hemipteran suborders was immediately followed by a Triassic diversification of heteropteran infraorders (figure 4). From a Carboniferous origin, early terrestrial lineages of Hemiptera radiated soon after the hypothesized origin of gymnosperms [47], and formed Sternorrhyncha, Auchenorrhyncha, Coleorrhyncha, and Heteroptera in the Permian.

With the exception of some mycophagous nymphs, Sternorrhyncha, Auchenorrhyncha, and Coleorrhyncha are entirely phytophagous, feeding on fluids of phloem, xylem, or cambium, with some inducing galls (some Psylloidea, Aphidoidea, and Coccoidea) [21]. Extinct hemipteran taxa that formed the ancestral stock of today's major lineages were consistently linked to gymnosperms. Shcherbakov [48] inferred ‘such short-legged Archescytinidae (primitive Hemiptera) either lived in confined spaces of gymnosperm reproductive organs or clung tightly to the plant surface’. Small, usually dorsoventrally depressed hoppers and their flattened cryptic nymphs (a body form possibly adapted to living between cone scales) [49] probably fed on phloem of thick gymnosperm stems [21]. The first xylem-feeding Hemiptera existed in the gymnosperm-dominated Permian and Triassic forests, while the large and clumsily built early Permian boreoscytids possibly fed on large gymnosperm ovules [21]. Furthermore, fossils representing the early members of Sternorrhyncha and Auchenorrhyncha were recovered from the same Kungurian beds (275–270 Ma), coincident with gymnosperm dominance [21]. However, most family-level diversification events in extant Sternorrhyncha seem to coincide with the angiosperm radiation, as indicated in our analysis (Psylloidea may be exceptional in that all eight extant families are not older than the Eocene) [50]. Ortiz-Rivas *et al*. [51] likewise linked angiosperm and aphid tribe diversification, producing angiosperm-feeding taxa. As all extant superfamilies of Sternorrhyncha (scales, aphids, whiteflies) feed on angiosperms and gymnosperms, yet evolved from gymnosperm feeders [17,51–53], it is difficult to deduce the finer mechanisms governing their evolution. This notion is especially complicated considering that well after the era of gymnosperm replacement with angiosperms (beginning 150 Ma), there was an increase in gymnosperm diversification rates persisting over the last 100 Ma [54].

The evolution and diversification of seed plants give rise to vast ecological niches [46]. The evolution of the seed has not only promoted the evolutionary success of plants for nearly 400 Ma but also probably initiated and facilitated the subsequent success of Hemiptera. Among the four hemipteran suborders, Heteroptera displays the greatest diversity in their habitat and behaviour, as well as species diversity. The origin of Heteroptera (approx. 262 Ma; CI, 273–249 Ma) coincided with the evolution of the apically produced labium (electronic supplementary material, figure S1*d*; i.e. a gula permitting a prognathous rostrum position), predatory behaviour, and the novel protective forewing. The true bug infraorders diversified in the Late Permian and Triassic (262–226 Ma). We propose that the diversification of potential prey species following the P-T extinction (252 Ma) [55] may have paved the way for the diversification of the arthropod-feeding heteropteran lineages. Two key adaptations that facilitated the rapid family-level radiation of Heteroptera coincide with the shift from gymnosperm- to angiosperm-dominance. The evolution of prognathous mouthparts and the novel hemelytron probably facilitated multiple independent evolutions of predatory behaviour from a phytophagous ancestor and, consequently, multiple transitions to aquatic and semi-aquatic habitats. The prognathous mouthparts clearly facilitated the development of a more versatile suite of feeding behaviours including predation, blood feeding, and mycophagy, none of which occurs in the other three predominantly phytophagous and entirely terrestrial suborders. This behavioural diversity may explain the higher rates of diversification (of extant lineages) in Heteroptera, composing more number of families and species than the other three hemipteran suborders.

Angiosperm coevolution is often the default explanation for major radiations. The family diversifications of Sternorrhyncha (without Psylloidea) and Pentatomomorpha coincide, in large part, with the consequent decline of gymnosperms (i.e. shift to angiosperm dominance; 125–100 Ma) [56]. However, at the family-level, there is little clear association to be made between the radiations of hemipterans and angiosperms, probably because the latter is much older than the former as in the case of Psylloidea. Although angiosperms may have driven familial or intrafamilial diversity in many groups, the diversity of habitat and feeding behaviour observed in Heteroptera can almost entirely be linked to diversification events coincident with an era of angiosperm suppression before 150 Ma. Of course, hypothesizing explanations for such ancient events remains challenging. Future studies focusing on thorough sampling of each suborder/infraorder must be conducted to elucidate finer intrafamilial radiation stories (perhaps for interfamilial relationships as well).

Similarly, Hunt *et al*. [4] failed to directly link the ‘superradiation’ of beetles (which account for 25% of all metazoans) at 285 Ma with angiosperm coevolution. Like beetles, true bugs exhibit immense versatility with diverse habitat colonization, varied feeding habits, and modified forewings that confer protection [57] and even facilitate plastron (air bubble) retention for aquatic respiration. The modified, protective forewings of beetles and true bugs may account for the rapid lineage diversification and probably facilitated the versatile feeding and habitat colonization, including multiple independent shifts to predation and aquatic habitats [4] that gave rise to the biodiversity before us today. The radiation of angiosperms may have simply facilitated an already bustling process.

## Supplementary Material

Hu Li_figures_ESM.pdf

## Supplementary Material

Hu Li_tables_ESM.xlsx

## Data accessibility

The annotated mitogenome sequences of 34 hemipteran insects have been deposited in GenBank (accession numbers shown in electronic supplementary material, table S1). The data supporting this article have been uploaded as the electronic supplementary material and the datasets used in the phylogenetic analyses are available in the Dryad Digital Repository: http://dx.doi.org/10.5061/dryad.13m7r [58].
