# Supplementary material for: Mitochondrial phylogenomics of Hemiptera reveals adaptive innovations driving the diversification of true bugs
Source: Proc Biol Sci. 2017 Sep 6;284(1862):20171223. doi: 10.1098/rspb.2017.1223 (PMC5597834; doi:10.1098/rspb.2017.1223)
Supplement: Hu Li_figures_ESM.pdf [file rspb20171223supp1.pdf]

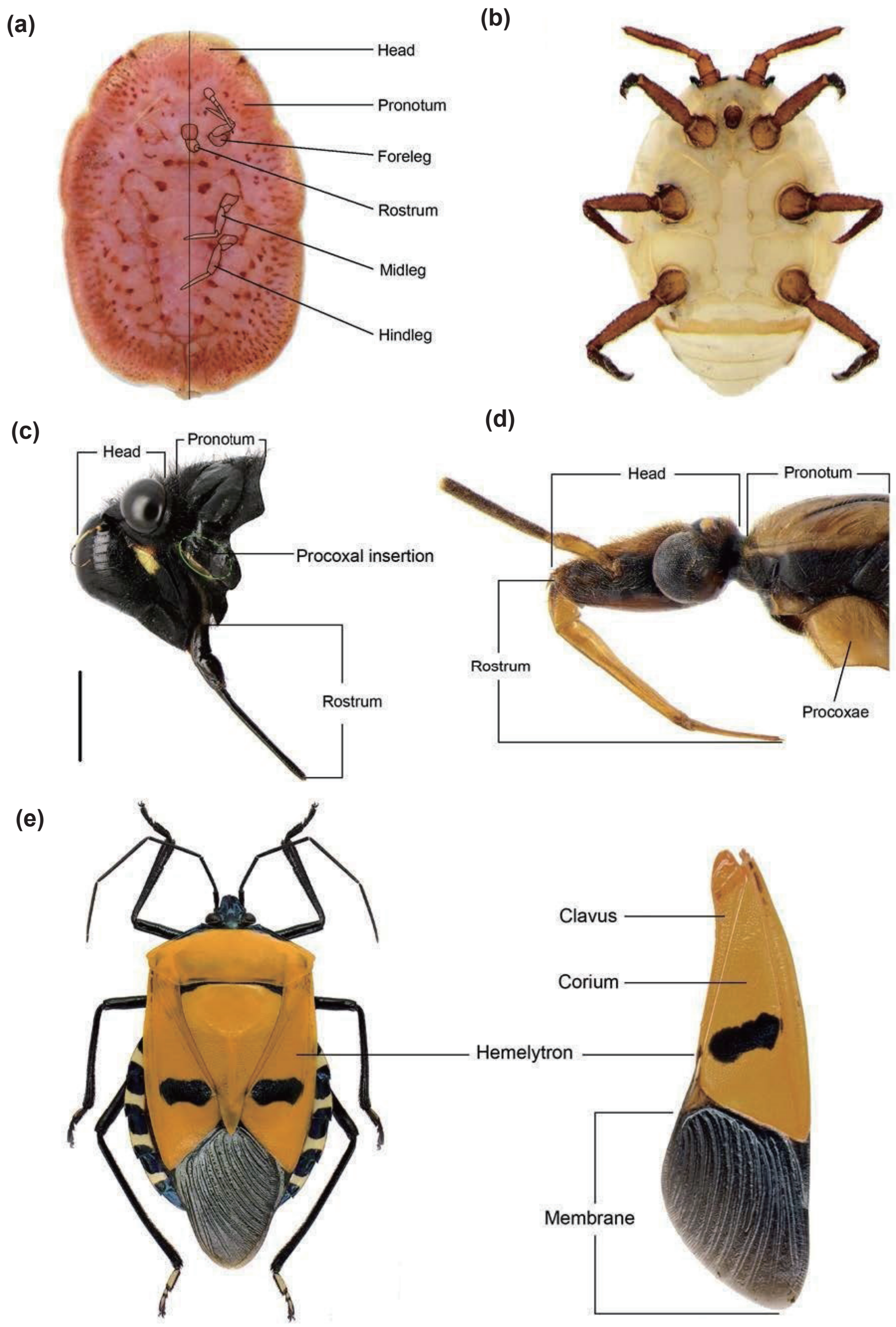

Fig. S1. Mouthpart origin and hemelytra of hemipteran insects. (*a*) Hypognathous mouthpart position (wax scale insect, *Ceroplastes* sp.). (*b*) *Orthezia* sp. (scale insect). (*c*) Orthognathous mouthpart position (cicada, *Gaeana maculate*). (*d*) Prognathous mouthpart position (assassin bug, *Sirthenea flavipes*). (*e*) Pentatomid bug, *Catacanthus incarnatus* showing hemelytron structure. Scale: for (*a*), 0.40 mm; for (*b*), 0.65 mm; for (*c*), 3.84 mm; for (*d*), 1.81 mm; for (*e*), 6.71 mm (for body of pentatomid bug) and 4.73 mm (for hemelytron).

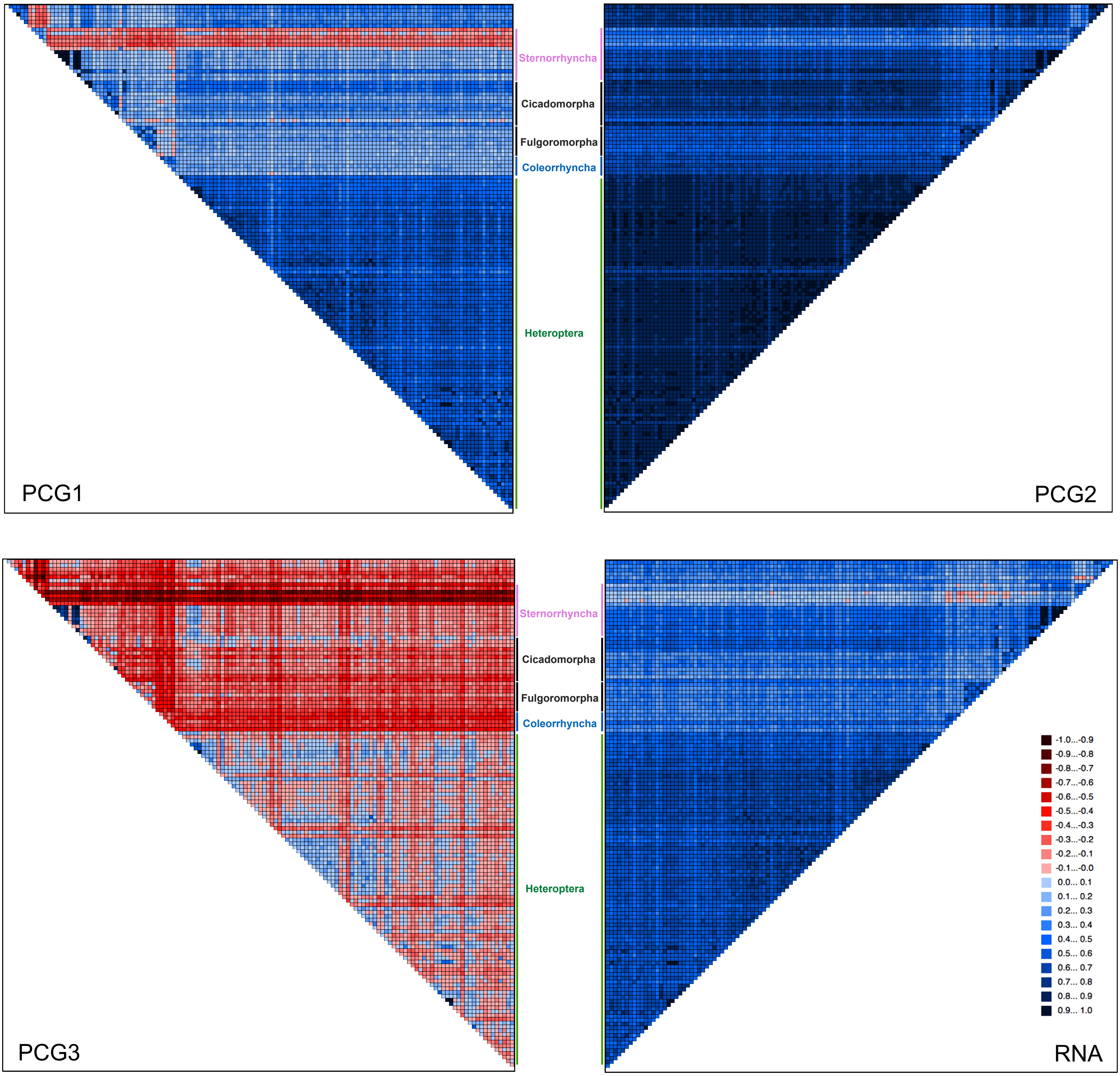

Fig. S2. AliGROOVE analysis for codon positions of protein-coding genes (PCGs) and RNA genes. PCG1, the first codon position of PCGs. PCG2, the second codon position of PCGs. PCG3, the third codon position of PCGs. RNA, sequences of tRNA and rRNA genes. The mean similarity score between sequences is represented by a colored square, based on AliGROOVE scores from -1, indicating great difference in rates from the remainder of the data set, that is, heterogeneity (red coloring), to +1, indicating that ratesmatch all other comparisons (blue coloring).

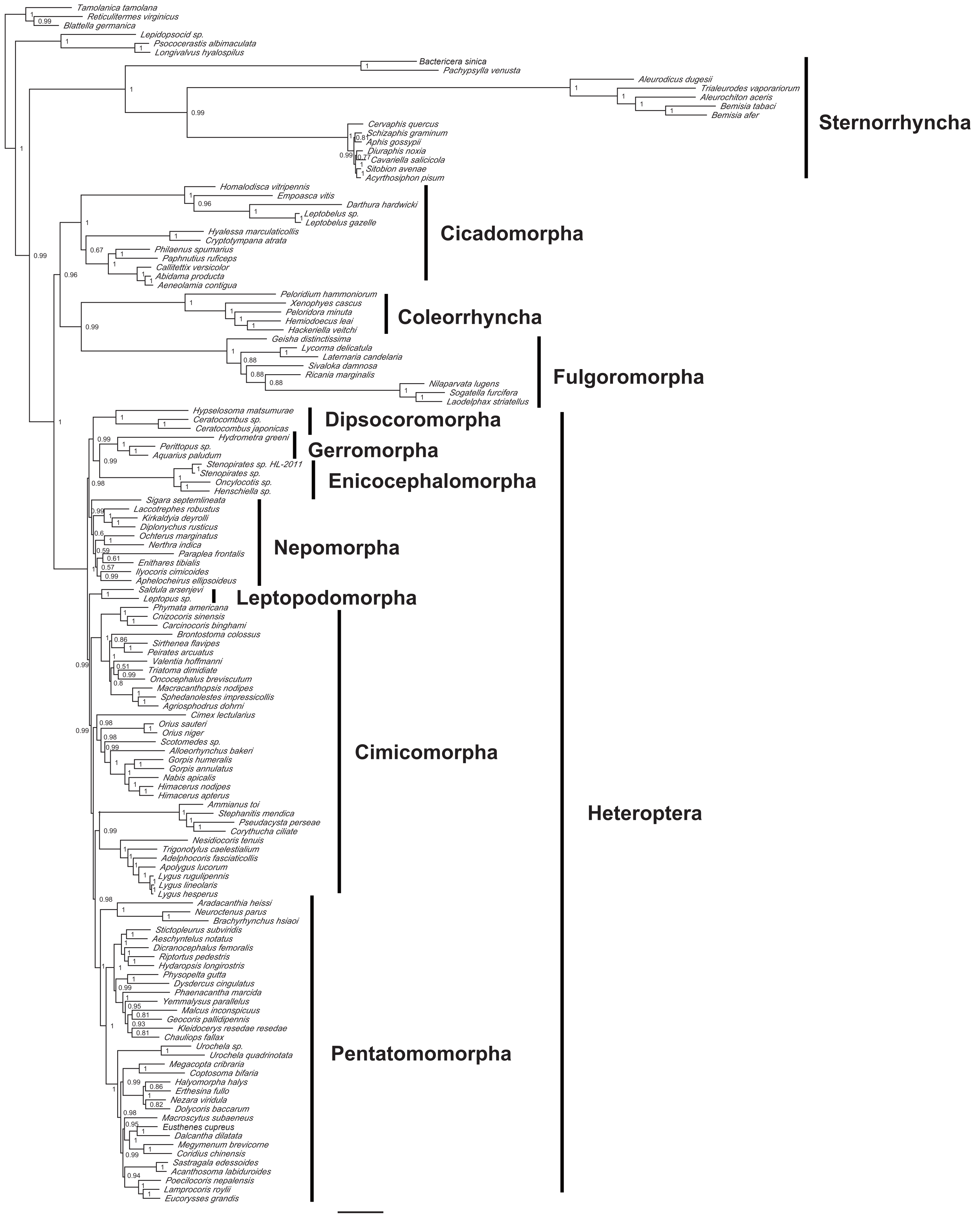

Fig. S3. Phylogenetic tree inferred from PhyloBayes analysis of the PCGRNA dataset under the CAT+GTR mixture model. Values at nodes are Bayesian PPs.

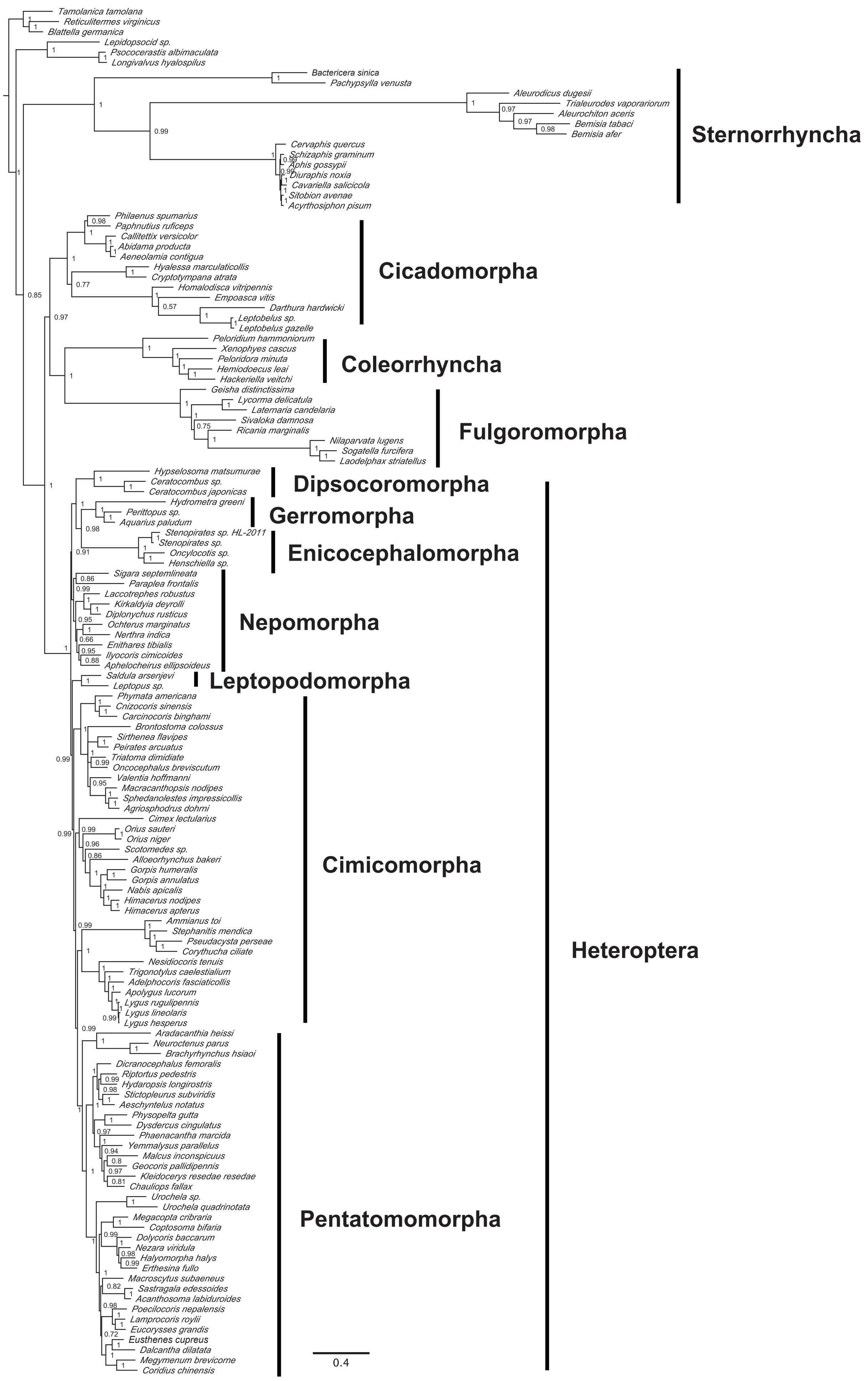

Fig. S4. Phylogenetic tree inferred from PhyloBayes analysis of the PCG12RNA dataset under the CAT+GTR mixture model. Values at nodes are Bayesian PPs.

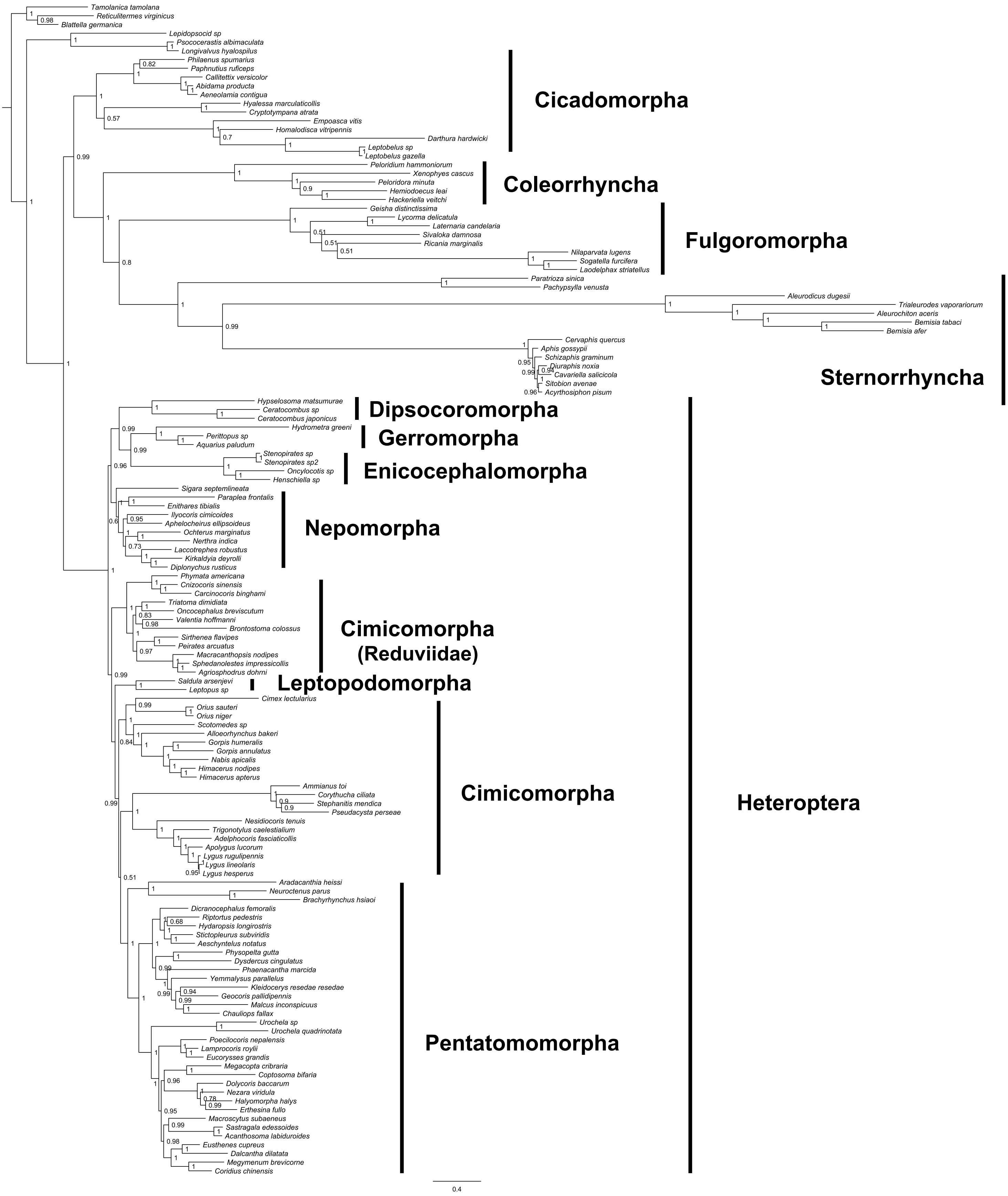

Fig. S5. Phylogenetic tree inferred from PhyloBayes analysis of the AA dataset under the CAT+GTR mixture model. Values at nodes are Bayesian PPs.

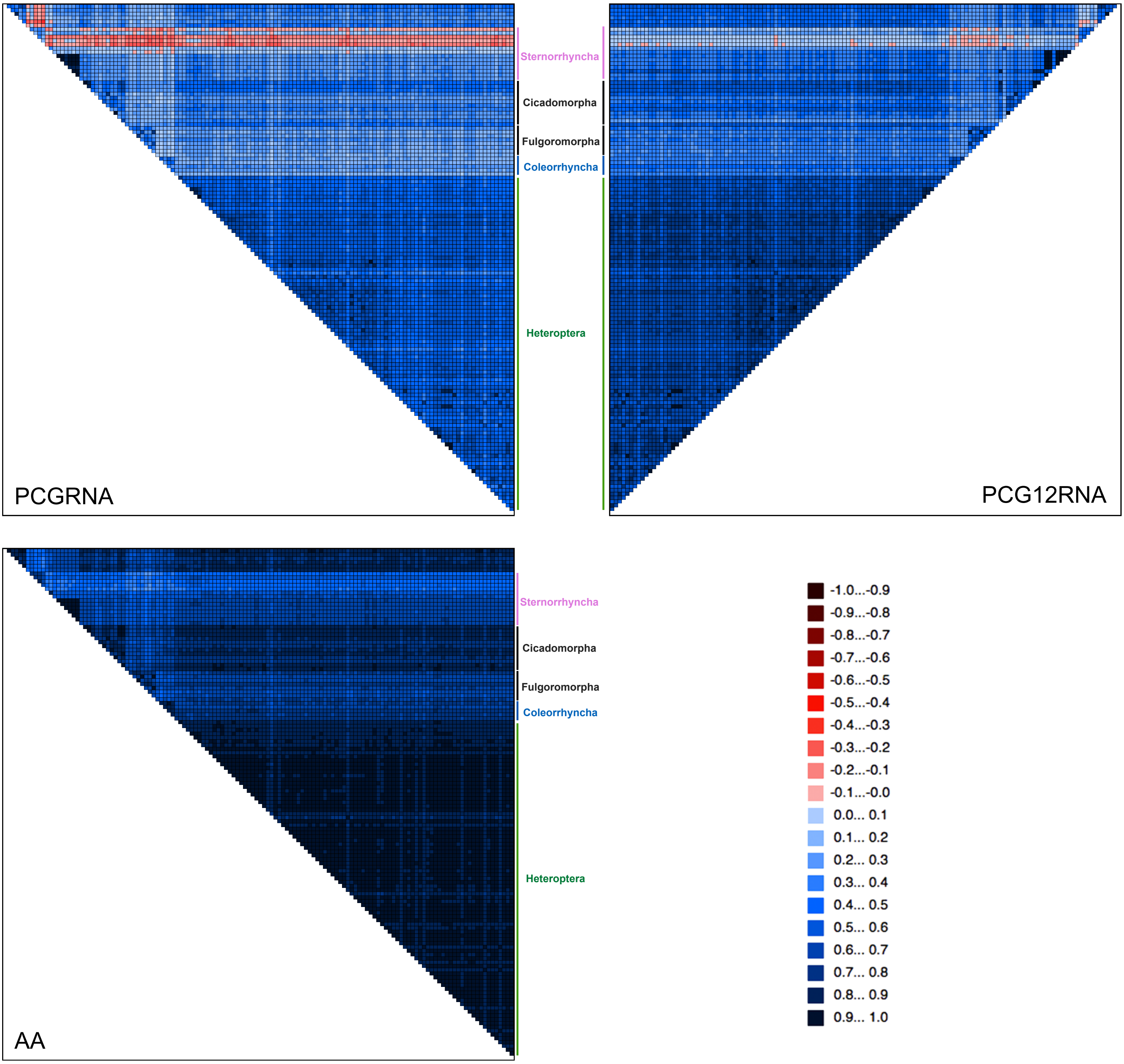

Fig. S6. AliGROOVE analysis for PCG12RNA, PCGRNA and AA datasets. The mean similarity score between sequences is represented by a colored square, based on AliGROOVE scores from -1, indicating great difference in rates from the remainder of the data set, that is, heterogeneity (red coloring), to +1, indicating that ratesmatch all other comparisons (blue coloring).

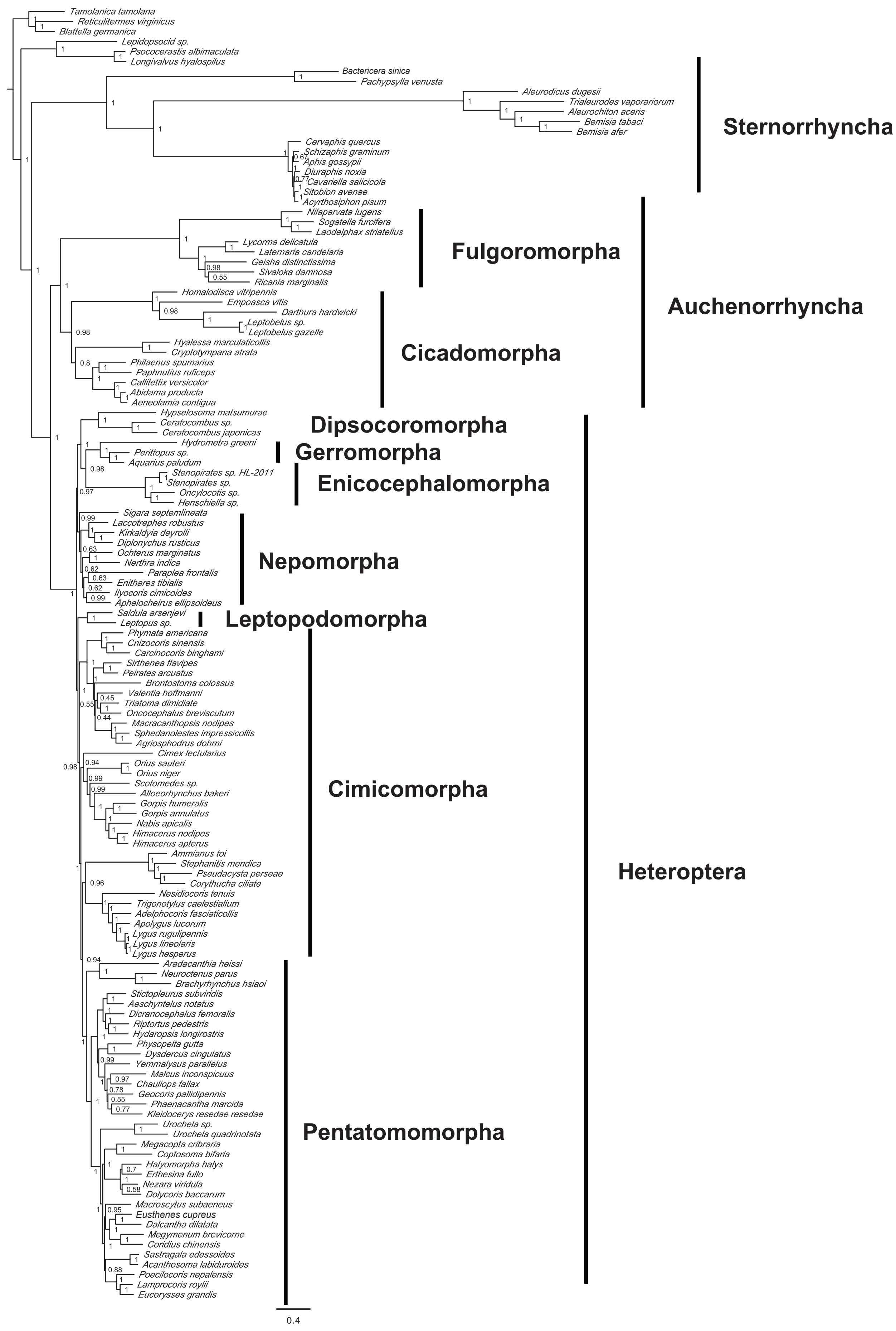

Fig. S7. Phylogenetic tree inferred from PhyloBayes analysis of the PCGRNA dataset under the CAT +GTR mixture model. Species from Coleorrhyncha are excluded in the analysis. Values at nodes are Bayesian PPs.

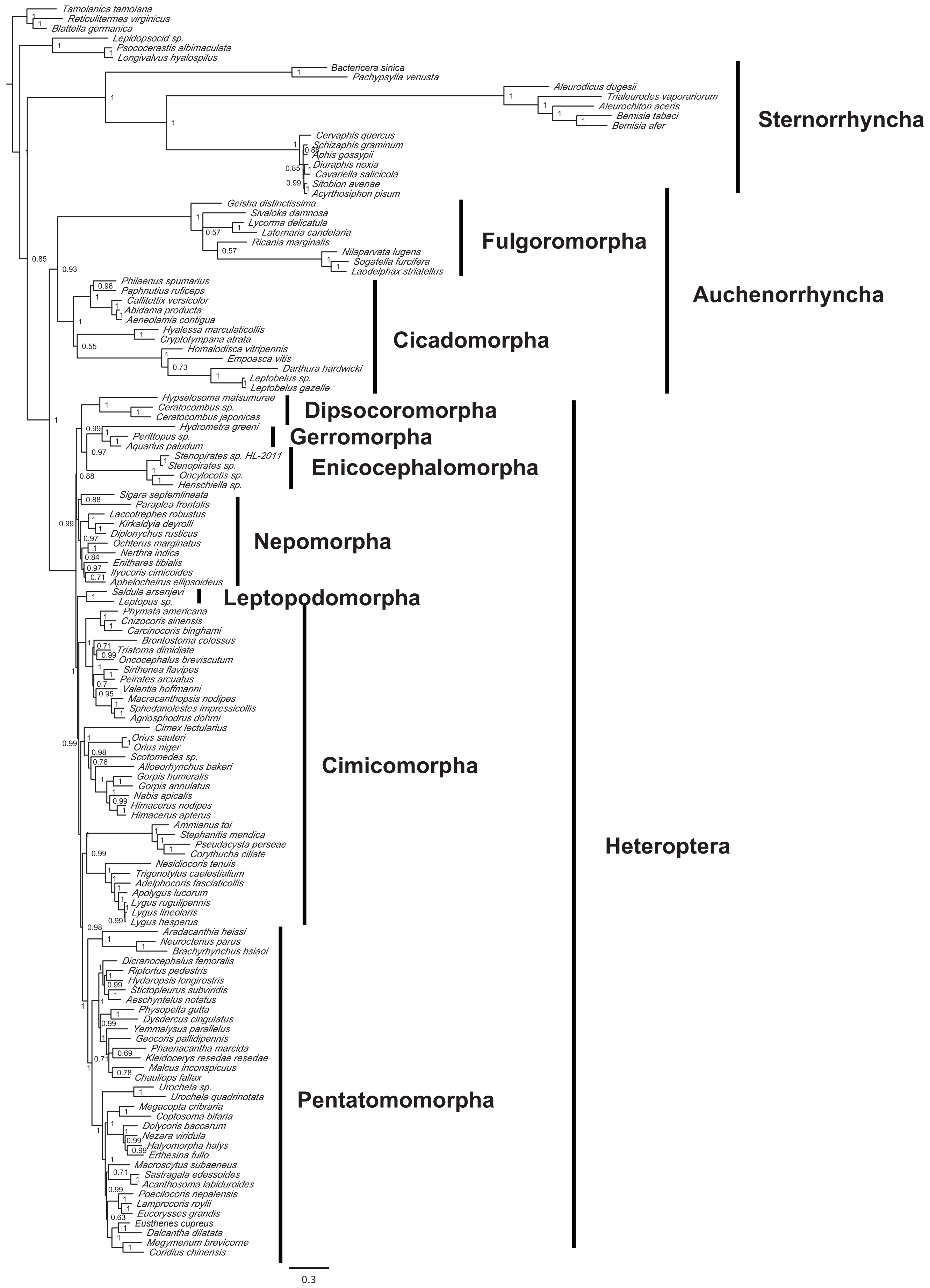

Fig. S8. Phylogenetic tree inferred from PhyloBayes analysis of the PCG12RNA dataset under the CAT+GTR mixture model. Species from Coleorrhyncha are excluded in the analysis. Values at nodes are Bayesian PPs.

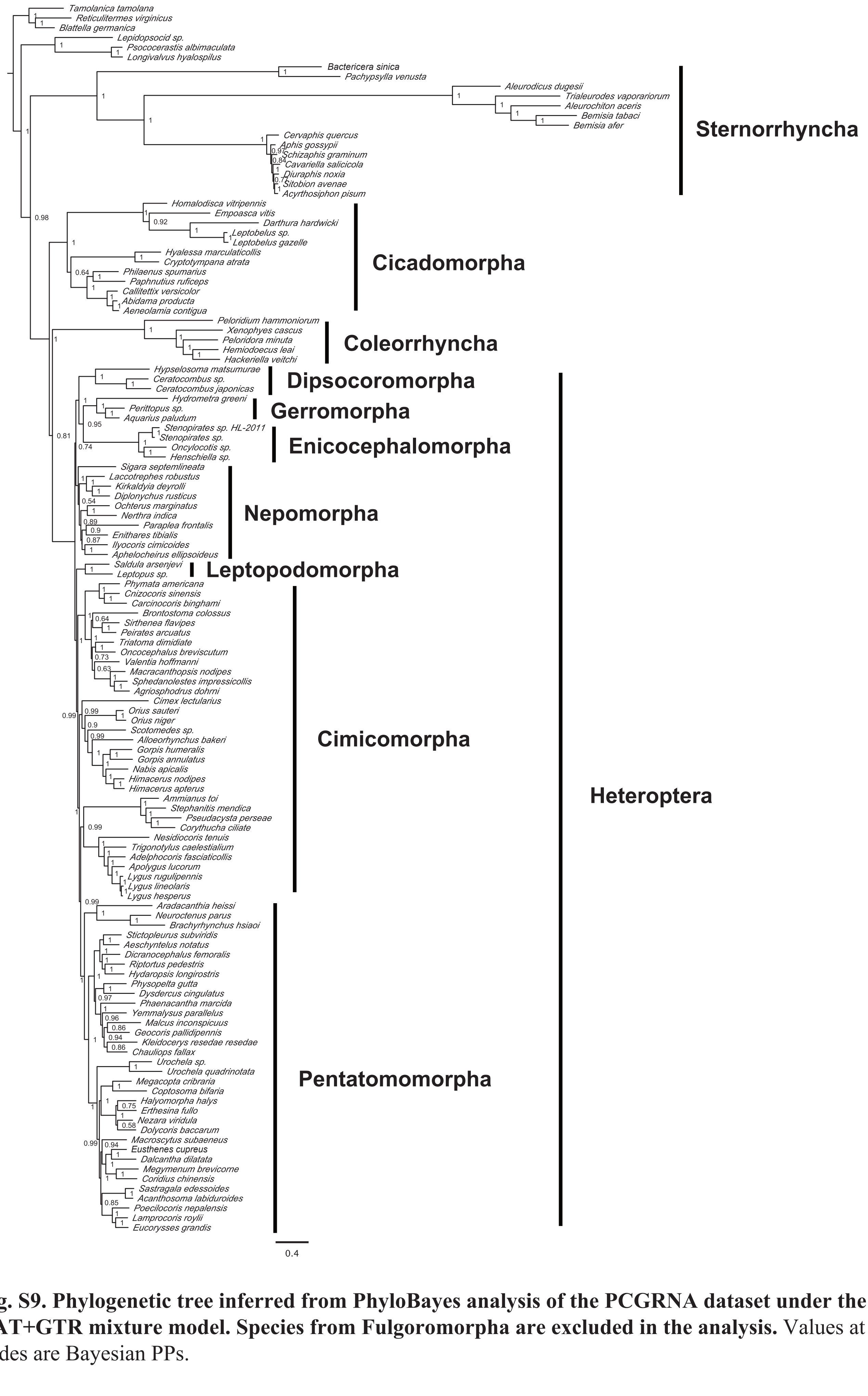

Fig. S9. Phylogenetic tree inferred from PhyloBayes analysis of the PCGRNA dataset under the CAT+GTR mixture model. Species from Fulgoromorpha are excluded in the analysis. Values at nodes are Bayesian PPs.

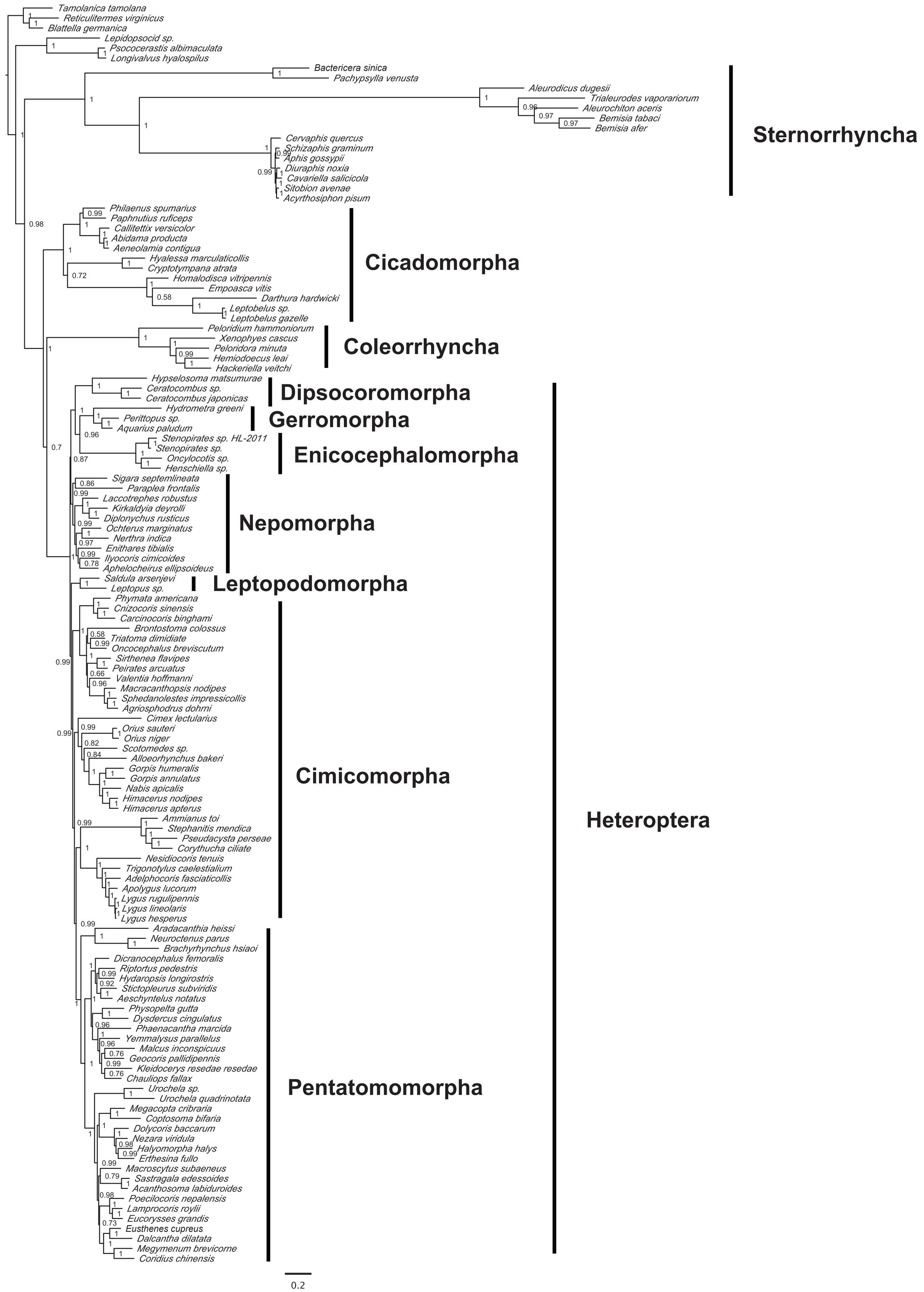

Fig. S10. Phylogenetic tree inferred from PhyloBayes analysis of the PCG12RNA dataset under the CAT+GTR mixture model. Species from Fulgoromorpha are excluded in the analysis. Values at nodes are Bayesian PPs.

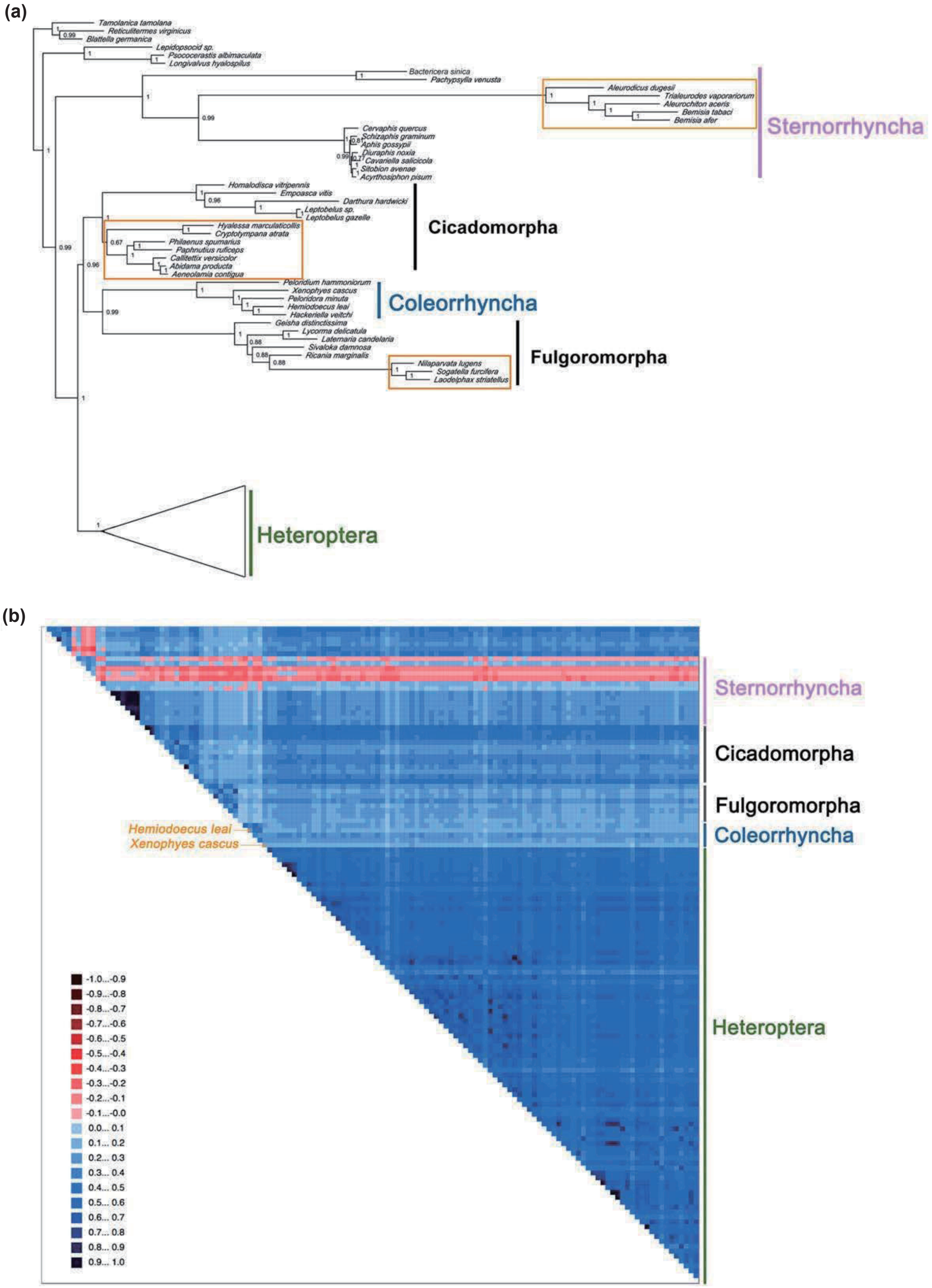

Fig. S11. The improved taxon sampling from Sternorrhyncha, Fulgoromorpha, Cicadomorpha and Coleorrhyncha. (*a*) Five species with longest branches in Sternorrhyncha, seven species with short branches in Cicadomorpha and three species with longest branches in Fulgoromorpha, were excluded according to terminal branches in the phylogenetic tree inferred from Bayesian analysis of the PCGRNA dataset. The excluded species were highlighted with orange. (*b*) Two moss bugs (Coleorrhyncha) with strong heterogeneity in sequence divergence were excluded according to the AliGROOVE analysis of the PCGRNA dataset. The obtained mean similarity score between sequences is represented by a colored square. The scores range from -1, indicating full random similarity, to +1, indicating non-random similarity. The darker red indicates the higher randomized accordance between pairwise sequence comparisons. Blue indicates the opposite.

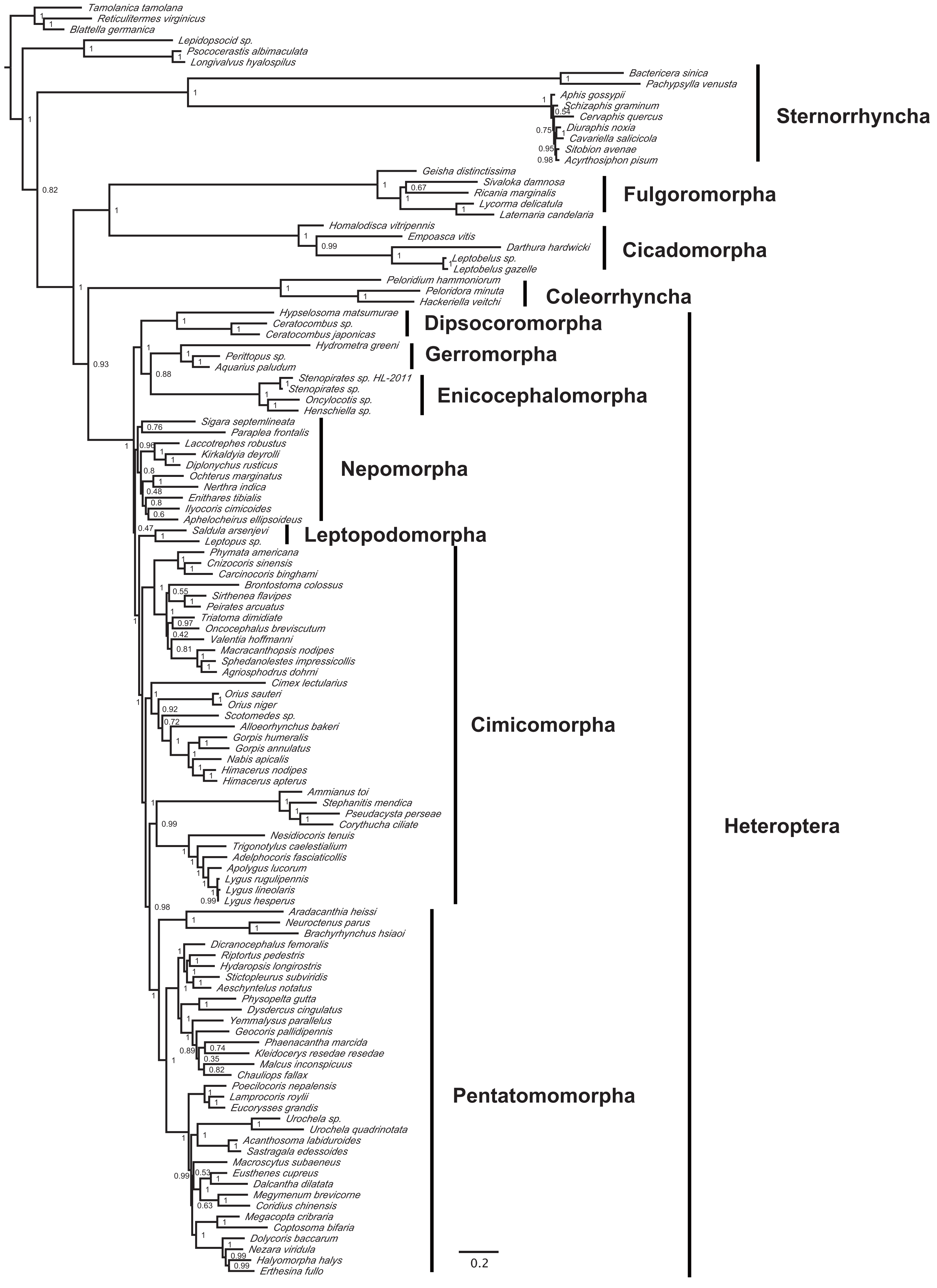

Fig. S12. Phylogenetic tree inferred from PhyloBayes analysis of the PCG12RNA dataset (117-taxa) under the CAT+GTR mixture model. Values at nodes are Bayesian PPs.

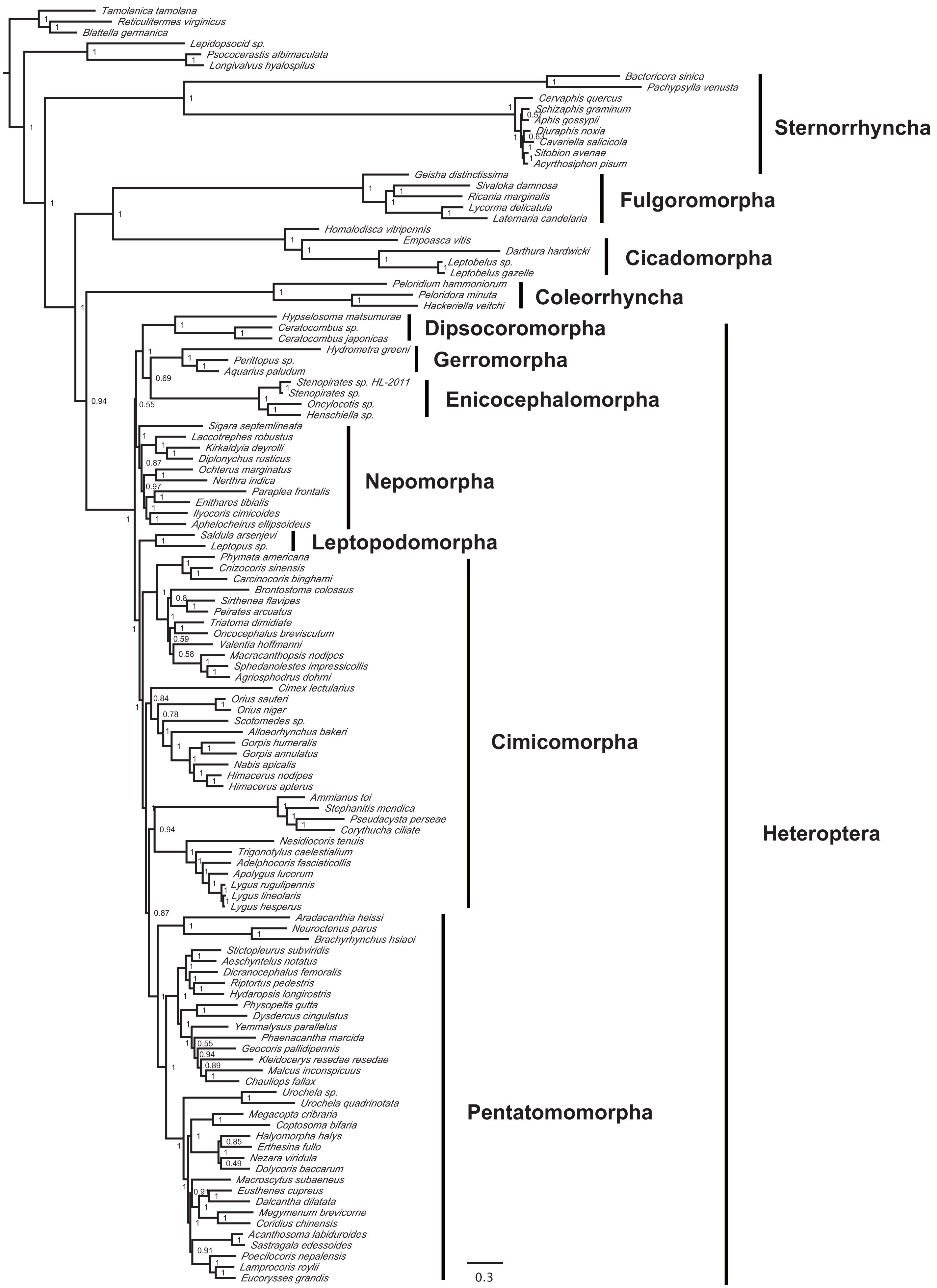

Fig. S13. Phylogenetic tree inferred from PhyloBayes analysis of the PCGRNA dataset (117-taxa) under the CAT+GTR mixture model. Values at nodes are Bayesian PPs.

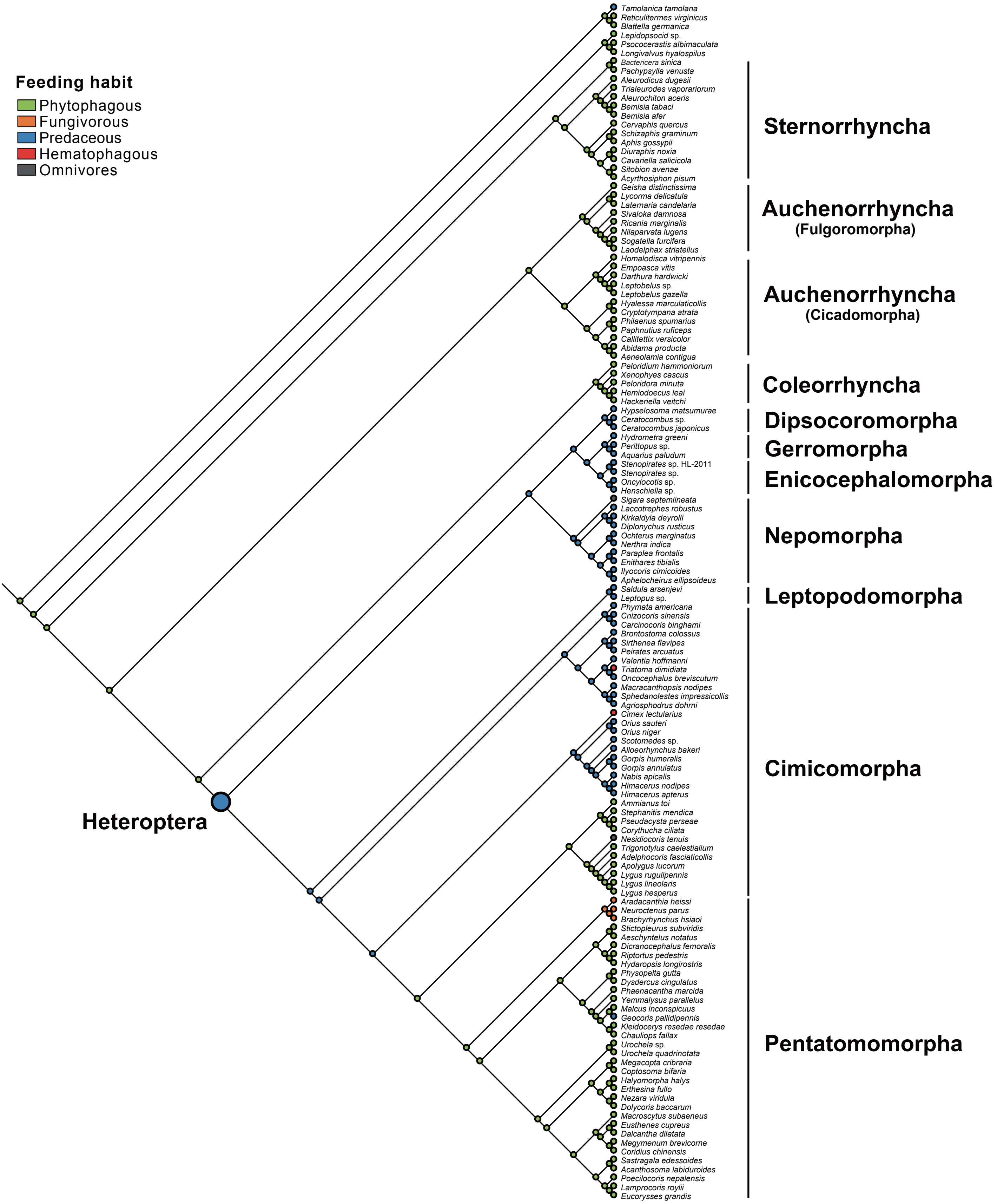

Fig. S14. ML optimization of feeding habit conducted with Mesquite under the MK1 model of character evolution.

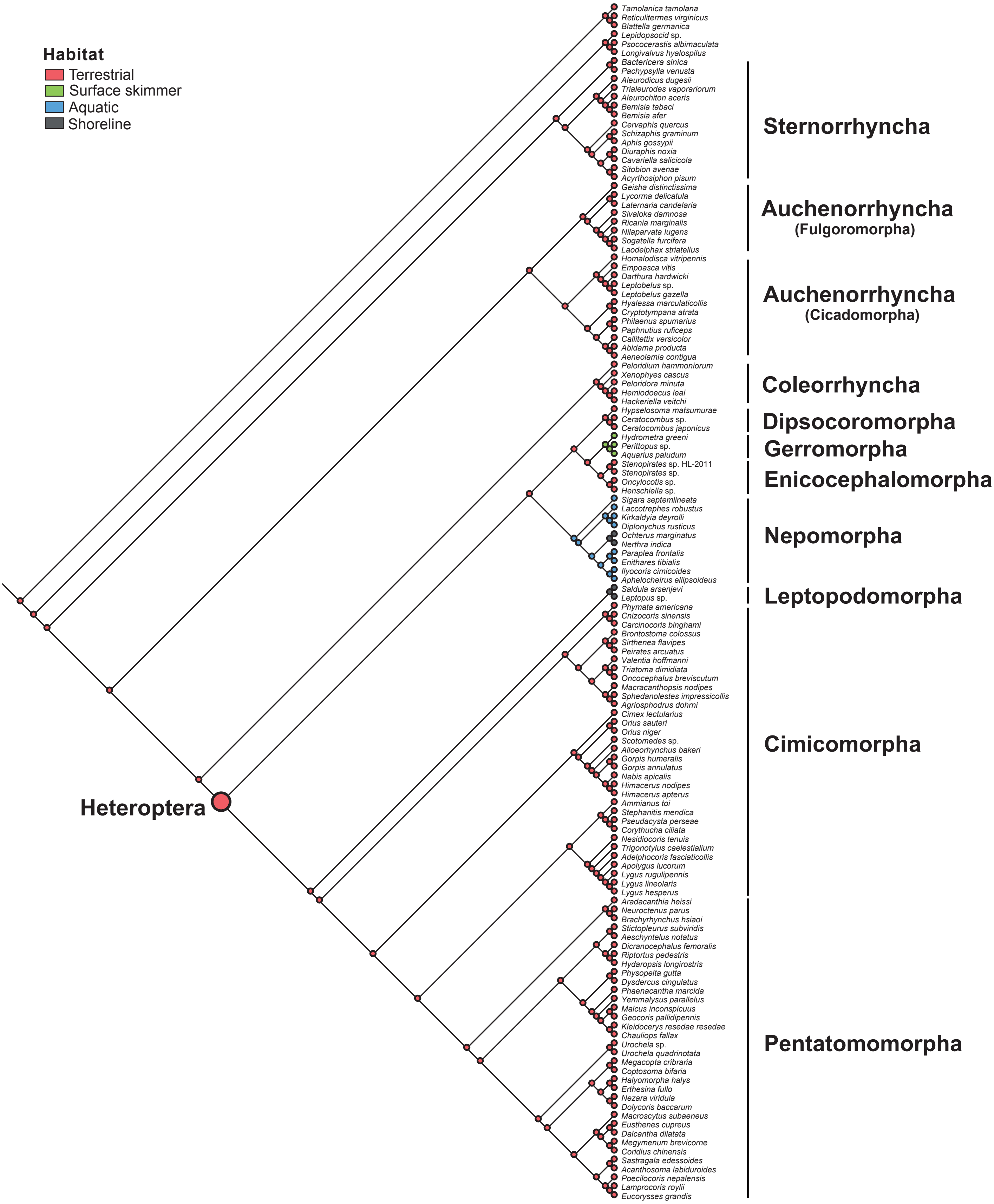

Fig. S15. ML optimization of habitat conducted with Mesquite under the MK1 model of character evolution.

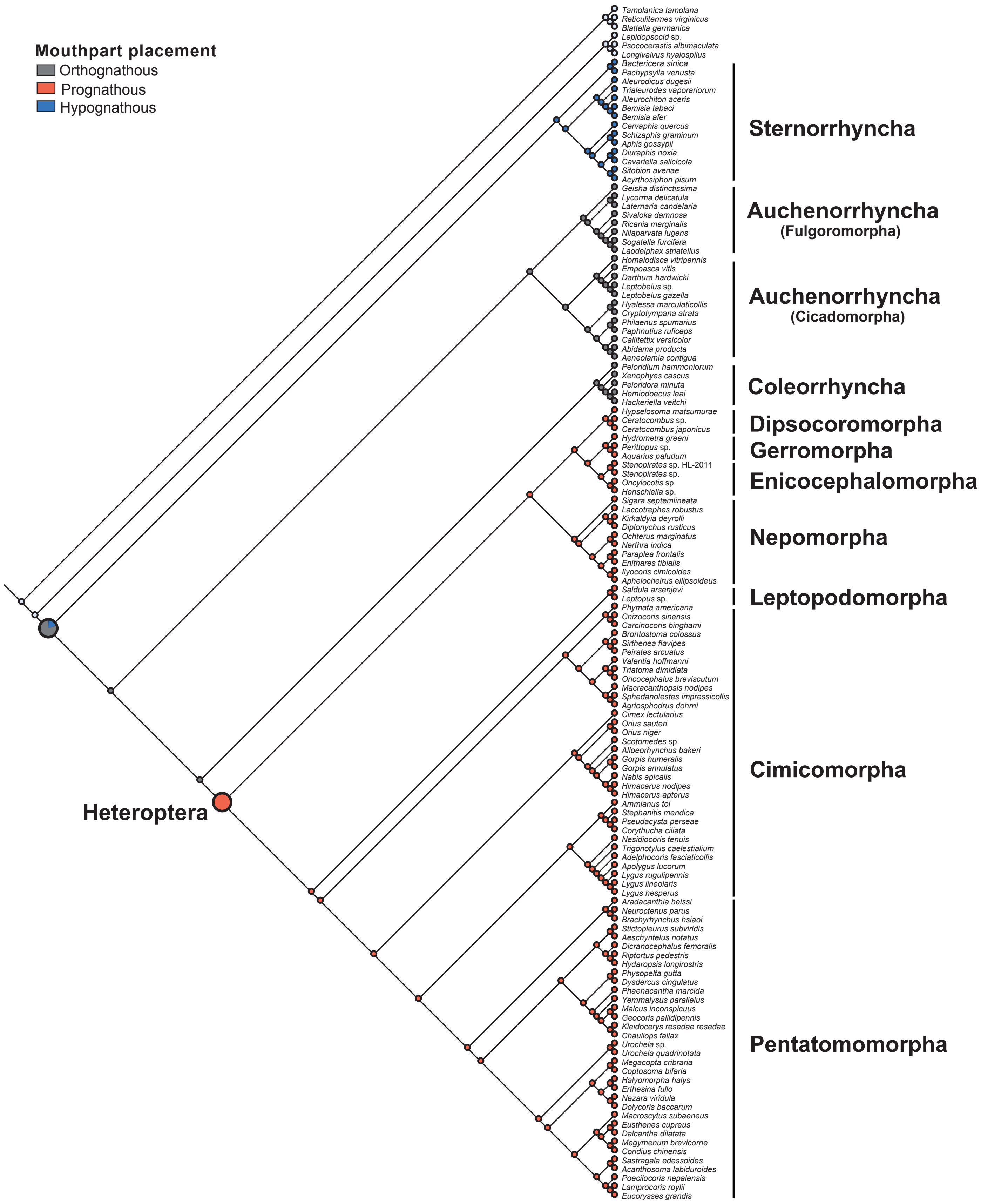

Fig. S16. ML optimization of mouthpart placement conducted with Mesquite under the MK1 model of character evolution.

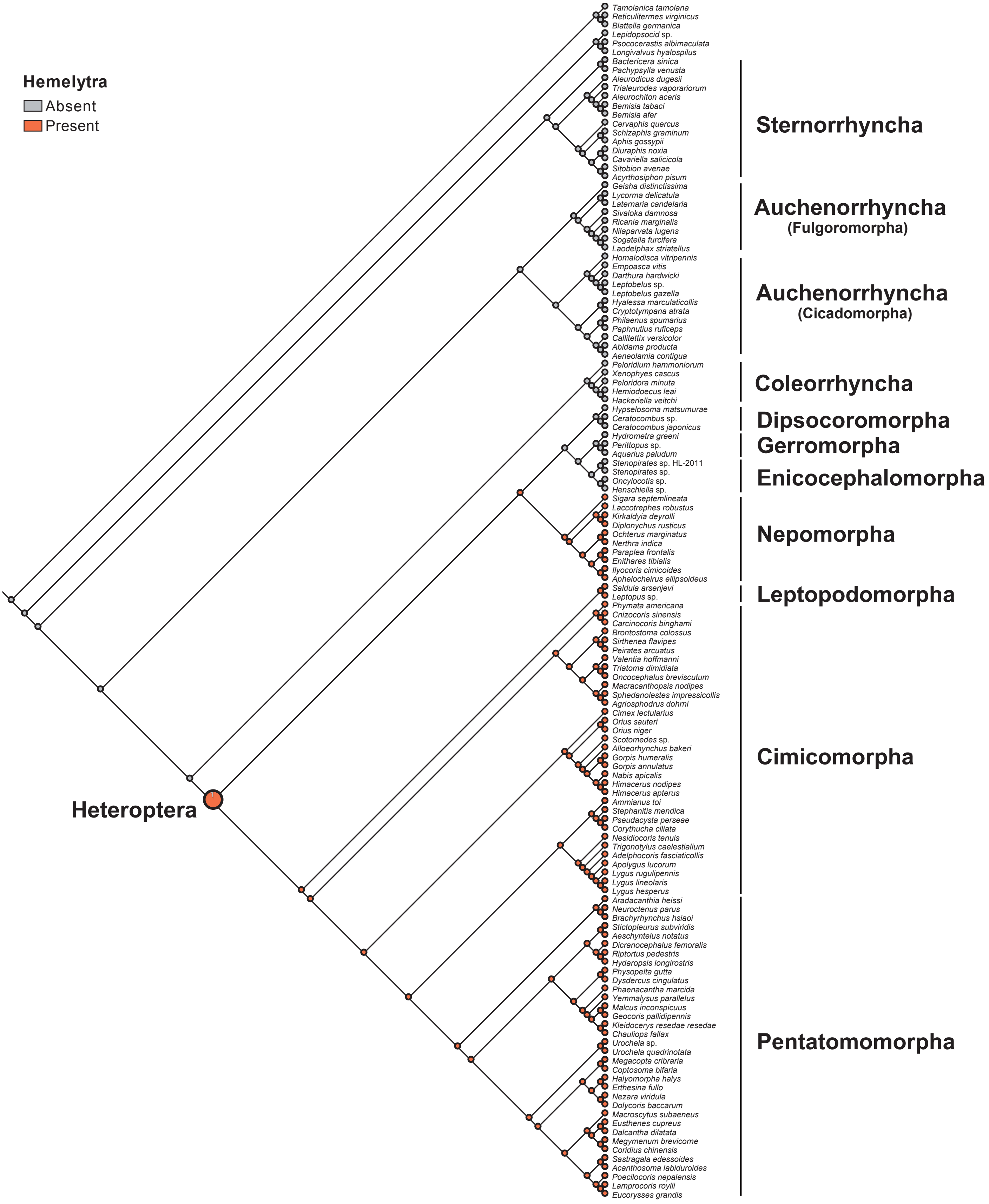

Fig. S17. ML optimization of hemeltra conducted with Mesquite under the MK1 model of character evolution.
